# Monoclinic form of (*Z*)-1-ferrocenyl-3-(3-hy­droxy­anilino)but-2-en-1-one

**DOI:** 10.1107/S1600536811047775

**Published:** 2011-11-16

**Authors:** Jie Zhou, Yuan-Yuan Ji, Yao-Cheng Shi, Wen-Bin Shen, Li-Min Yuan

**Affiliations:** a Department of Chemical and Biological Engineering, Nantong Vocational College, Nantong 226007, People’s Republic of China; b College of Chemistry and Chemical Engineering, Yangzhou University, Yangzhou 225002, People’s Republic of China; c Analytical Center, China Pharmaceutical University, Nanjin 210009, People’s Republic of China; d Testing Center, Yangzhou University, Yangzhou 225009, People’s Republic of China

## Abstract

The title compound, [Fe(C_5_H_5_)(C_15_H_14_NO_2_)], is a monoclinic polymorph of the previously reported triclinic form [Shi *et al.* (2006 ▶). *Acta Cryst*. C**62**, m407–m410]. The polymorphs feature the same strong intra­molecular N—H⋯O=C hydrogen bonds, but show different packing modes. The mol­ecules in the monoclinic form associate into double chains *via* O—H⋯O=C and (Cp)C—H⋯O—H inter­actions.

## Related literature

For background to enamino­nes in coordination chemistry, supra­molecular chemistry, organometallic chemistry and organic synthesis, see: Shi *et al.* (2004 ▶, 2005 ▶, 2006 ▶, 2008 ▶); Shi & Hu (2009 ▶); Elassar & El-Khair (2003 ▶); Kascheres (2003 ▶). For related structures, see: Shi & Zhang (2007 ▶).
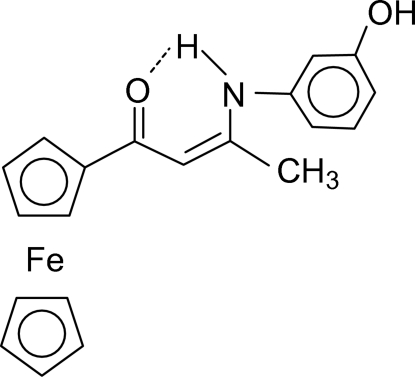


## Experimental

### 

#### Crystal data

[Fe(C_5_H_5_)(C_15_H_14_NO_2_)]
*M* *_r_* = 361.21
Monoclinic, 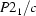

*a* = 8.4123 (17) Å
*b* = 13.1124 (11) Å
*c* = 16.2327 (14) Å
β = 103.931 (3)°
*V* = 1737.9 (4) Å^3^
*Z* = 4
Mo *K*α radiation
μ = 0.88 mm^−1^
*T* = 296 K
0.21 × 0.17 × 0.11 mm

#### Data collection

Bruker SMART APEX CCD diffractometer
Absorption correction: multi-scan (*SADABS*; Sheldrick, 2004 ▶) *T*_min_ = 0.828, *T*_max_ = 0.902
14899 measured reflections
4008 independent reflections
1999 reflections with *I* > 2σ(*I*)
*R*_int_ = 0.086

#### Refinement

*R*[*F* ^2^ > 2σ(*F* ^2^)] = 0.048
*wR*(*F* ^2^) = 0.106
*S* = 0.97
4008 reflections
227 parameters
40 restraints
H atoms treated by a mixture of independent and constrained refinement
Δρ_max_ = 0.32 e Å^−3^
Δρ_min_ = −0.35 e Å^−3^

### 

Data collection: *SMART* (Bruker, 2002 ▶); cell refinement: *SAINT-Plus* (Bruker, 2003 ▶); data reduction: *SAINT-Plus*; program(s) used to solve structure: *SIR2004* (Burla *et al.*, 2005 ▶); program(s) used to refine structure: *SHELXTL* (Sheldrick, 2008 ▶); molecular graphics: *PLATON* (Spek, 2009 ▶) and *WinGX* (Farrugia, 1999 ▶); software used to prepare material for publication: *publCIF* (Westrip, 2010 ▶).

## Supplementary Material

Crystal structure: contains datablock(s) I, global. DOI: 10.1107/S1600536811047775/tk5015sup1.cif

Structure factors: contains datablock(s) I. DOI: 10.1107/S1600536811047775/tk5015Isup2.hkl

Additional supplementary materials:  crystallographic information; 3D view; checkCIF report

## Figures and Tables

**Table 1:** Hydrogen-bond geometry (Å, °)

*D*—H⋯*A*	*D*—H	H⋯*A*	*D*⋯*A*	*D*—H⋯*A*
N1—H1*N*⋯O1	0.85 (3)	1.94 (3)	2.632 (3)	139 (3)
O2—H2⋯O1^i^	0.87 (4)	1.79 (4)	2.662 (3)	178 (5)
C12—H12⋯O2^ii^	0.93	2.46	3.281 (4)	148

Symmetry codes: (i) 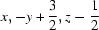; (ii) 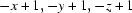.

**Fig. 1.**
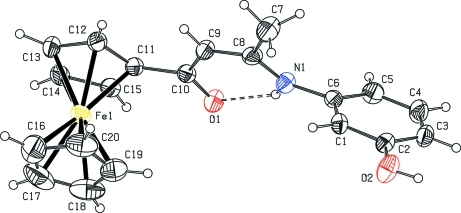
The molecule of the title compound, showing the atom-labelling scheme. Displacement ellipsoids are drawn at the 30% probability level.

## supplementary crystallographic information

### Comment

Recently, enaminones and related compounds have been used as ligands in coordination chemistry (Shi *et al.*, 2004; Shi *et al.*, 2005; Shi *et al.*,  2008; Shi & Hu, 2009) and have been extensively used as versatile synthetic intermediates that combine the variable nucleophilicity of enamines with the variable electrophilicity of enones for the preparation of a variety of heterocyclic systems including some natural products and analogues (Elassar & El-Khair, 2003; Kascheres, 2003). It has also been shown that primary amines, Ar'NH_2_, react smoothly with β–diketones, ArCOCH_2_COR, to give enaminones, ArCOCH═C(NHAr')*R*, in good yields (Shi *et al.*, 2004; Shi & Zhang, 2007). As part of an on-going investigation of the chemistry of enaminones and related compounds (Shi *et al.*, 2005; Shi *et al.*, 2006), the title compound has been synthesized *via* the reaction of 3–H_2_NC_6_H_4_OH and FcCOCH_2_COCH_3_ (Fig. 1).

The title compound crystallizes in two polymorphic forms I and II. The form I belongs to the space group *P*2_1_/*c* with *Z*' = 1 while the form II previously reported crystallises in the triclinic space group P1 with *Z*' = 2 (Shi *et al.*, 2006).

As noted for form II, having two different conformations for the molecule (IIa and IIb) in the asymmetric unit, the C—C═C—N moiety in the monoclinic polymorph is planar and the bond lengths indicate electron delocalization (Shi *et al.*, 2004), Table 1. The O═C—C═C—N plane is twisted with respect to the benzene and substituted cyclopentadienyl rings by 59.89 (16) and 6.21 (18)°, respectively, whereas the corresponding values in the form II are 70.5 (2) and 21.0 (3)° for IIa and 66.4 (2) and 19.5 (3)° for IIb.

Although the two polymorphs each have the strong N—H···O═C intramolecular hydrogen bonds, they show different packing modes. The molecules in the form I form dimers by C—H(Cp)···O—H (1 - *x*, 1 - *y*, 1 - *z*) hydrogen bonds, thus resulting in [001] double-chains *via* O—H···O═ C (*x*, -*y* + 3/2, *z* - 1/2) hydrogen bonds, Table 2. However, the molecules in the form II associate *via* O—H(IIa)···O═ C(IIb) (*x* - 1, *y* + 1, *z*) hydrogen bonds to generate [110] chains which are linked by O—H(IIb)···O═C(IIa) hydrogen bonds.

### Experimental

A solution of ferrocenoylacetone (1.35 g, 5 mmol) and 3–aminophenol (0.55 g, 5 mmol) in anhydrous ethanol (25 ml) was refluxed overnight. After removal of the solvent, the resulting solid was purified by chromatography on silica gel with dichloromethane as eluant to give a red solid. Recrystallization from ethanol solution affords bright red single crystals of the title compound. *M*.pt. 482.65–483.35 K. IR (KBr): 3418 (w, OH), 3047 (m, NH), 1587 (*vs*, O═C), 1524 (m, C═C) cm^-1. 1^H NMR (600 MHz, CDCl_3_, δ, p.p.m.): 12.595 (s, 1H, NH), 8.646 (s, br, 1H, OH), 7.22, 6.84, 6.79, 6.68 (t, *J* = 7.8 Hz, 1H, s, 1H, d, *J* = 9.6 Hz, 1H, d, *J* = 7.8 Hz, 1H, C_6_H_4_), 5.48 (s, 1H, CH), 4.82, 4.44 (s, s, 2H, 2H, C_5_H_4_), 4.20 (s, 5H, C_5_H_5_), 2.10 (s, 3H, CH_3_). UV (in DMF ,λ_max_ (ε×10^4^)): 265 (4.91), 360.00 (2.04), 550.00 (0.018) nm.

### Refinement

The H atoms attached to the N and O atoms were refined freely. The remaining H atoms were placed at geometrically idealized positions and subsequently treated as riding with C—H = 0.93–0.98 Å, and with *U*_iso_(H) = 1.2*U*_eq_(C) or 1.5*U*_eq_(C_methyl_).

### Crystal data

**Table:**

[Fe(C_5_H_5_)(C_15_H_14_NO_2_)]	*F*(000) = 752
*M**_r_* = 361.21	*D*_x_ = 1.380 Mg m^−^^3^
Monoclinic, *P*2_1_/*c*	Mo *K*α radiation, λ = 0.71073 Å
Hall symbol: -P 2ybc	Cell parameters from 1999 reflections
*a* = 8.4123 (17) Å	θ = 2.5–27.7°
*b* = 13.1124 (11) Å	µ = 0.88 mm^−^^1^
*c* = 16.2327 (14) Å	*T* = 296 K
β = 103.931 (3)°	Prism, red
*V* = 1737.9 (4) Å^3^	0.21 × 0.17 × 0.11 mm
*Z* = 4	

### Data collection

**Table:**

Bruker SMART APEX CCD diffractometer	4008 independent reflections
Radiation source: fine-focus sealed tube	1999 reflections with *I* > 2σ(*I*)
graphite	*R*_int_ = 0.086
ω and φ scans	θ_max_ = 27.7°, θ_min_ = 2.5°
Absorption correction: multi-scan (*SADABS*; Sheldrick, 2004)	*h* = −10→10
*T*_min_ = 0.828, *T*_max_ = 0.902	*k* = −17→16
14899 measured reflections	*l* = −21→21

### Refinement

**Table:**

Refinement on *F*^2^	Primary atom site location: structure-invariant direct methods
Least-squares matrix: full	Secondary atom site location: difference Fourier map
*R*[*F*^2^ > 2σ(*F*^2^)] = 0.048	Hydrogen site location: inferred from neighbouring sites
*wR*(*F*^2^) = 0.106	H atoms treated by a mixture of independent and constrained refinement
*S* = 0.97	*w* = 1/[σ^2^(*F*_o_^2^) + (0.0378*P*)^2^] where *P* = (*F*_o_^2^ + 2*F*_c_^2^)/3
4008 reflections	(Δ/σ)_max_ = 0.001
227 parameters	Δρ_max_ = 0.32 e Å^−^^3^
40 restraints	Δρ_min_ = −0.35 e Å^−^^3^

### Special details

**Table:**

Geometry. All e.s.d.'s (except the e.s.d. in the dihedral angle between two l.s. planes) are estimated using the full covariance matrix. The cell e.s.d.'s are taken into account individually in the estimation of e.s.d.'s in distances, angles and torsion angles; correlations between e.s.d.'s in cell parameters are only used when they are defined by crystal symmetry. An approximate (isotropic) treatment of cell e.s.d.'s is used for estimating e.s.d.'s involving l.s. planes.
Refinement. Refinement of *F*^2^ against ALL reflections. The weighted *R*-factor *wR* and goodness of fit *S* are based on *F*^2^, conventional *R*-factors *R* are based on *F*, with *F* set to zero for negative *F*^2^. The threshold expression of *F*^2^ > σ(*F*^2^) is used only for calculating *R*-factors(gt) *etc*. and is not relevant to the choice of reflections for refinement. *R*-factors based on *F*^2^ are statistically about twice as large as those based on *F*, and *R*- factors based on ALL data will be even larger.

### Fractional atomic coordinates and isotropic or equivalent isotropic displacement parameters (Å^2^)

**Table:**

	*x*	*y*	*z*	*U*_iso_*/*U*_eq_	
C1	0.7390 (4)	0.6856 (2)	0.49623 (19)	0.0445 (8)	
H1	0.6384	0.6524	0.4823	0.053*	
C2	0.7741 (4)	0.7611 (2)	0.44342 (19)	0.0446 (8)	
C3	0.9248 (4)	0.8081 (2)	0.4627 (2)	0.0548 (9)	
H3	0.9501	0.8573	0.4266	0.066*	
C4	1.0374 (4)	0.7818 (3)	0.5358 (2)	0.0649 (10)	
H4	1.1389	0.8140	0.5491	0.078*	
C5	1.0030 (4)	0.7084 (2)	0.5901 (2)	0.0566 (9)	
H5	1.0799	0.6920	0.6398	0.068*	
C6	0.8537 (4)	0.6598 (2)	0.56964 (19)	0.0421 (8)	
C7	0.7495 (5)	0.4441 (2)	0.5305 (2)	0.0815 (13)	
H7A	0.8528	0.4546	0.5164	0.122*	
H7B	0.6635	0.4732	0.4869	0.122*	
H7C	0.7304	0.3723	0.5347	0.122*	
C8	0.7521 (4)	0.4949 (2)	0.61447 (19)	0.0490 (8)	
C9	0.7000 (4)	0.4432 (2)	0.67679 (18)	0.0479 (8)	
H9	0.6647	0.3763	0.6655	0.057*	
C10	0.6959 (4)	0.4844 (2)	0.75694 (18)	0.0428 (8)	
C11	0.6275 (4)	0.4230 (2)	0.81607 (17)	0.0414 (7)	
C12	0.5473 (4)	0.3266 (2)	0.79989 (19)	0.0487 (8)	
H12	0.5349	0.2886	0.7504	0.058*	
C13	0.4899 (4)	0.2989 (3)	0.8715 (2)	0.0555 (9)	
H13	0.4289	0.2367	0.8774	0.067*	
C14	0.5328 (4)	0.3771 (3)	0.9327 (2)	0.0531 (9)	
H14	0.5066	0.3785	0.9883	0.064*	
C15	0.6166 (4)	0.4536 (2)	0.89952 (18)	0.0471 (8)	
H15	0.6604	0.5168	0.9284	0.056*	
C16	0.1421 (5)	0.4183 (4)	0.7808 (4)	0.1025 (15)	
H16	0.0836	0.3583	0.7817	0.123*	
C17	0.1800 (6)	0.4919 (5)	0.8443 (3)	0.1110 (17)	
H17	0.1510	0.4888	0.8960	0.133*	
C18	0.2659 (6)	0.5690 (4)	0.8194 (3)	0.1009 (14)	
H18	0.3045	0.6271	0.8506	0.121*	
C19	0.2856 (5)	0.5455 (3)	0.7395 (3)	0.0854 (12)	
H19	0.3403	0.5852	0.7076	0.102*	
C20	0.2104 (5)	0.4533 (4)	0.7149 (3)	0.0878 (13)	
H20	0.2057	0.4200	0.6638	0.105*	
Fe1	0.39001 (6)	0.43521 (4)	0.82226 (3)	0.05174 (18)	
H2	0.687 (5)	0.832 (3)	0.344 (3)	0.116 (17)*	
H1N	0.822 (4)	0.611 (2)	0.6781 (19)	0.052 (10)*	
N1	0.8147 (3)	0.5887 (2)	0.62841 (18)	0.0497 (8)	
O1	0.7493 (3)	0.57348 (16)	0.77857 (12)	0.0550 (6)	
O2	0.6531 (3)	0.7862 (2)	0.37474 (16)	0.0698 (8)	

### Atomic displacement parameters (Å^2^)

**Table:**

	*U*^11^	*U*^22^	*U*^33^	*U*^12^	*U*^13^	*U*^23^
C1	0.045 (2)	0.0471 (19)	0.0433 (19)	−0.0100 (16)	0.0137 (16)	−0.0027 (16)
C2	0.058 (2)	0.0401 (19)	0.0356 (18)	−0.0031 (17)	0.0112 (17)	−0.0010 (15)
C3	0.062 (2)	0.050 (2)	0.056 (2)	−0.0069 (19)	0.021 (2)	0.0103 (18)
C4	0.044 (2)	0.061 (2)	0.088 (3)	−0.0108 (19)	0.013 (2)	0.013 (2)
C5	0.047 (2)	0.062 (2)	0.056 (2)	0.0013 (19)	0.0020 (18)	0.0052 (19)
C6	0.049 (2)	0.0406 (18)	0.0406 (19)	0.0021 (16)	0.0175 (16)	−0.0013 (16)
C7	0.145 (4)	0.055 (2)	0.061 (2)	−0.005 (2)	0.059 (3)	−0.011 (2)
C8	0.062 (2)	0.043 (2)	0.046 (2)	0.0069 (18)	0.0200 (17)	−0.0016 (16)
C9	0.065 (2)	0.0394 (18)	0.0423 (19)	−0.0038 (17)	0.0201 (16)	−0.0023 (16)
C10	0.044 (2)	0.0466 (19)	0.0385 (18)	0.0041 (16)	0.0108 (15)	0.0023 (16)
C11	0.0468 (19)	0.0440 (19)	0.0342 (17)	0.0031 (16)	0.0112 (14)	0.0013 (15)
C12	0.053 (2)	0.048 (2)	0.046 (2)	0.0014 (17)	0.0141 (17)	0.0007 (16)
C13	0.055 (2)	0.053 (2)	0.062 (2)	−0.0012 (18)	0.0206 (19)	0.0153 (19)
C14	0.057 (2)	0.064 (2)	0.0436 (19)	0.0107 (18)	0.0209 (18)	0.0125 (19)
C15	0.050 (2)	0.052 (2)	0.0399 (18)	0.0072 (17)	0.0113 (16)	0.0026 (16)
C16	0.046 (3)	0.109 (4)	0.142 (4)	−0.001 (2)	0.001 (3)	0.042 (3)
C17	0.072 (3)	0.166 (5)	0.101 (3)	0.053 (3)	0.032 (3)	0.025 (3)
C18	0.087 (3)	0.097 (3)	0.104 (3)	0.042 (3)	−0.007 (3)	−0.012 (3)
C19	0.072 (3)	0.085 (3)	0.093 (3)	0.026 (2)	0.007 (2)	0.034 (3)
C20	0.073 (3)	0.100 (3)	0.076 (3)	0.027 (3)	−0.009 (2)	−0.001 (3)
Fe1	0.0456 (3)	0.0622 (3)	0.0485 (3)	0.0088 (3)	0.0135 (2)	0.0096 (3)
N1	0.066 (2)	0.0486 (19)	0.0371 (16)	−0.0015 (14)	0.0180 (15)	0.0002 (15)
O1	0.0757 (17)	0.0481 (14)	0.0440 (13)	−0.0111 (13)	0.0200 (12)	−0.0074 (11)
O2	0.0808 (19)	0.0705 (18)	0.0472 (15)	−0.0197 (15)	−0.0061 (14)	0.0150 (14)

### Geometric parameters (Å, °)

**Table:**

C1—C6	1.383 (4)	C12—H12	0.9300
C1—C2	1.387 (4)	C13—C14	1.413 (4)
C1—H1	0.9300	C13—Fe1	2.054 (3)
C2—O2	1.356 (4)	C13—H13	0.9800
C2—C3	1.376 (4)	C14—C15	1.405 (4)
C3—C4	1.372 (4)	C14—Fe1	2.049 (3)
C3—H3	0.9300	C14—H14	0.9800
C4—C5	1.382 (4)	C15—Fe1	2.028 (3)
C4—H4	0.9300	C15—H15	0.9800
C5—C6	1.376 (4)	C16—C17	1.392 (6)
C5—H5	0.9300	C16—C20	1.406 (6)
C10—O1	1.270 (3)	C16—Fe1	2.043 (4)
C6—N1	1.428 (4)	C16—H16	0.9300
C7—C8	1.513 (4)	C17—C18	1.359 (6)
C7—H7A	0.9600	C17—Fe1	2.026 (4)
C7—H7B	0.9600	C17—H17	0.9300
C7—H7C	0.9600	C18—C19	1.381 (6)
C8—N1	1.336 (4)	C18—Fe1	2.036 (4)
C8—C9	1.374 (4)	C18—H18	0.9300
C9—C10	1.417 (4)	C19—C20	1.378 (5)
C9—H9	0.9300	C19—Fe1	2.025 (4)
C10—C11	1.473 (4)	C19—H19	0.9300
C11—C12	1.426 (4)	C20—Fe1	2.026 (4)
C11—C15	1.437 (4)	C20—H20	0.9300
C11—Fe1	2.031 (3)	N1—H1N	0.85 (3)
C12—C13	1.410 (4)	O2—H2	0.88 (4)
C12—Fe1	2.035 (3)		
			
C6—C1—C2	119.9 (3)	C16—C17—Fe1	70.6 (3)
C6—C1—H1	120.0	C18—C17—H17	125.1
C2—C1—H1	120.0	C16—C17—H17	125.1
O2—C2—C3	123.1 (3)	Fe1—C17—H17	125.0
O2—C2—C1	116.9 (3)	C17—C18—C19	107.9 (5)
C3—C2—C1	120.0 (3)	C17—C18—Fe1	70.1 (3)
C4—C3—C2	119.4 (3)	C19—C18—Fe1	69.7 (2)
C4—C3—H3	120.3	C17—C18—H18	126.1
C2—C3—H3	120.3	C19—C18—H18	126.1
C3—C4—C5	121.3 (3)	Fe1—C18—H18	125.7
C3—C4—H4	119.4	C20—C19—C18	108.4 (5)
C5—C4—H4	119.4	C20—C19—Fe1	70.2 (2)
C6—C5—C4	119.2 (3)	C18—C19—Fe1	70.5 (3)
C6—C5—H5	120.4	C20—C19—H19	125.8
C4—C5—H5	120.4	C18—C19—H19	125.8
C5—C6—C1	120.1 (3)	Fe1—C19—H19	125.1
C5—C6—N1	119.0 (3)	C19—C20—C16	108.0 (4)
C1—C6—N1	120.7 (3)	C19—C20—Fe1	70.1 (2)
C8—C7—H7A	109.5	C16—C20—Fe1	70.4 (2)
C8—C7—H7B	109.5	C19—C20—H20	126.0
H7A—C7—H7B	109.5	C16—C20—H20	126.0
C8—C7—H7C	109.5	Fe1—C20—H20	125.1
H7A—C7—H7C	109.5	C19—Fe1—C17	66.3 (2)
H7B—C7—H7C	109.5	C19—Fe1—C20	39.78 (15)
N1—C8—C9	120.8 (3)	C17—Fe1—C20	66.9 (2)
N1—C8—C7	118.5 (3)	C19—Fe1—C15	121.24 (17)
C9—C8—C7	120.6 (3)	C17—Fe1—C15	125.2 (2)
C8—C9—C10	124.8 (3)	C20—Fe1—C15	155.92 (17)
C8—C9—H9	117.6	C19—Fe1—C11	107.37 (15)
C10—C9—H9	117.6	C17—Fe1—C11	161.4 (2)
O1—C10—C9	121.3 (3)	C20—Fe1—C11	120.36 (16)
O1—C10—C11	119.4 (3)	C15—Fe1—C11	41.47 (11)
C9—C10—C11	119.3 (3)	C19—Fe1—C12	125.32 (16)
C12—C11—C15	106.6 (3)	C17—Fe1—C12	157.0 (2)
C12—C11—C10	127.3 (3)	C20—Fe1—C12	108.09 (15)
C15—C11—C10	125.8 (3)	C15—Fe1—C12	68.82 (12)
C12—C11—Fe1	69.63 (17)	C11—Fe1—C12	41.07 (11)
C15—C11—Fe1	69.17 (17)	C19—Fe1—C18	39.77 (17)
C10—C11—Fe1	122.2 (2)	C17—Fe1—C18	39.09 (18)
C13—C12—C11	108.5 (3)	C20—Fe1—C18	66.89 (18)
C13—C12—Fe1	70.56 (18)	C15—Fe1—C18	108.21 (17)
C11—C12—Fe1	69.31 (17)	C11—Fe1—C18	124.91 (19)
C13—C12—H12	125.7	C12—Fe1—C18	161.90 (19)
C11—C12—H12	125.7	C19—Fe1—C16	67.26 (18)
Fe1—C12—H12	126.0	C17—Fe1—C16	40.01 (18)
C12—C13—C14	108.1 (3)	C20—Fe1—C16	40.44 (17)
C12—C13—Fe1	69.09 (17)	C15—Fe1—C16	161.68 (19)
C14—C13—Fe1	69.67 (18)	C11—Fe1—C16	155.96 (19)
C12—C13—H13	125.9	C12—Fe1—C16	121.41 (19)
C14—C13—H13	125.9	C18—Fe1—C16	67.0 (2)
Fe1—C13—H13	125.9	C19—Fe1—C14	156.23 (18)
C15—C14—C13	108.4 (3)	C17—Fe1—C14	109.52 (17)
C15—C14—Fe1	69.04 (17)	C20—Fe1—C14	162.59 (18)
C13—C14—Fe1	70.05 (18)	C15—Fe1—C14	40.30 (11)
C15—C14—H14	125.8	C11—Fe1—C14	68.73 (12)
C13—C14—H14	125.8	C12—Fe1—C14	68.08 (12)
Fe1—C14—H14	125.8	C18—Fe1—C14	121.98 (17)
C14—C15—C11	108.3 (3)	C16—Fe1—C14	125.79 (17)
C14—C15—Fe1	70.65 (19)	C19—Fe1—C13	162.09 (17)
C11—C15—Fe1	69.37 (17)	C17—Fe1—C13	122.88 (19)
C14—C15—H15	125.9	C20—Fe1—C13	125.95 (17)
C11—C15—H15	125.9	C15—Fe1—C13	68.10 (13)
Fe1—C15—H15	125.9	C11—Fe1—C13	68.61 (12)
C17—C16—C20	105.9 (5)	C12—Fe1—C13	40.35 (11)
C17—C16—Fe1	69.3 (3)	C18—Fe1—C13	156.67 (19)
C20—C16—Fe1	69.1 (2)	C16—Fe1—C13	108.86 (16)
C17—C16—H16	127.0	C14—Fe1—C13	40.28 (12)
C20—C16—H16	127.0	C8—N1—C6	129.3 (3)
Fe1—C16—H16	126.0	C8—N1—H1N	115 (2)
C18—C17—C16	109.8 (5)	C6—N1—H1N	116 (2)
C18—C17—Fe1	70.8 (3)	C2—O2—H2	111 (3)
			
C6—C1—C2—O2	−176.8 (3)	C11—C15—Fe1—C16	166.0 (5)
C6—C1—C2—C3	2.0 (4)	C11—C15—Fe1—C14	119.2 (3)
O2—C2—C3—C4	176.7 (3)	C14—C15—Fe1—C13	−37.14 (17)
C1—C2—C3—C4	−2.0 (5)	C11—C15—Fe1—C13	82.02 (18)
C2—C3—C4—C5	0.6 (5)	C12—C11—Fe1—C19	−124.3 (2)
C3—C4—C5—C6	0.9 (5)	C15—C11—Fe1—C19	117.8 (2)
C4—C5—C6—C1	−1.0 (5)	C10—C11—Fe1—C19	−2.2 (3)
C4—C5—C6—N1	−176.1 (3)	C12—C11—Fe1—C17	168.8 (5)
C2—C1—C6—C5	−0.5 (4)	C15—C11—Fe1—C17	50.9 (6)
C2—C1—C6—N1	174.6 (3)	C10—C11—Fe1—C17	−69.2 (6)
N1—C8—C9—C10	4.6 (5)	C12—C11—Fe1—C20	−82.9 (2)
C7—C8—C9—C10	−179.2 (3)	C15—C11—Fe1—C20	159.2 (2)
C8—C9—C10—O1	−3.4 (5)	C10—C11—Fe1—C20	39.2 (3)
C8—C9—C10—C11	176.2 (3)	C12—C11—Fe1—C15	117.9 (2)
O1—C10—C11—C12	173.1 (3)	C10—C11—Fe1—C15	−120.0 (3)
C9—C10—C11—C12	−6.5 (5)	C15—C11—Fe1—C12	−117.9 (2)
O1—C10—C11—C15	−1.1 (5)	C10—C11—Fe1—C12	122.0 (3)
C9—C10—C11—C15	179.3 (3)	C12—C11—Fe1—C18	−164.5 (2)
O1—C10—C11—Fe1	85.2 (3)	C15—C11—Fe1—C18	77.6 (2)
C9—C10—C11—Fe1	−94.4 (3)	C10—C11—Fe1—C18	−42.4 (3)
C15—C11—C12—C13	−0.3 (3)	C12—C11—Fe1—C16	−51.3 (4)
C10—C11—C12—C13	−175.5 (3)	C15—C11—Fe1—C16	−169.2 (4)
Fe1—C11—C12—C13	−59.9 (2)	C10—C11—Fe1—C16	70.7 (5)
C15—C11—C12—Fe1	59.5 (2)	C12—C11—Fe1—C14	80.61 (19)
C10—C11—C12—Fe1	−115.6 (3)	C15—C11—Fe1—C14	−37.31 (18)
C11—C12—C13—C14	0.2 (4)	C10—C11—Fe1—C14	−157.3 (3)
Fe1—C12—C13—C14	−58.9 (2)	C12—C11—Fe1—C13	37.22 (18)
C11—C12—C13—Fe1	59.1 (2)	C15—C11—Fe1—C13	−80.69 (19)
C12—C13—C14—C15	0.0 (4)	C10—C11—Fe1—C13	159.3 (3)
Fe1—C13—C14—C15	−58.5 (2)	C13—C12—Fe1—C19	−165.3 (2)
C12—C13—C14—Fe1	58.5 (2)	C11—C12—Fe1—C19	75.2 (3)
C13—C14—C15—C11	−0.2 (4)	C13—C12—Fe1—C17	−51.3 (5)
Fe1—C14—C15—C11	−59.4 (2)	C11—C12—Fe1—C17	−170.9 (4)
C13—C14—C15—Fe1	59.2 (2)	C13—C12—Fe1—C20	−124.7 (2)
C12—C11—C15—C14	0.4 (3)	C11—C12—Fe1—C20	115.8 (2)
C10—C11—C15—C14	175.6 (3)	C13—C12—Fe1—C15	80.7 (2)
Fe1—C11—C15—C14	60.2 (2)	C11—C12—Fe1—C15	−38.87 (17)
C12—C11—C15—Fe1	−59.8 (2)	C13—C12—Fe1—C11	119.6 (3)
C10—C11—C15—Fe1	115.4 (3)	C13—C12—Fe1—C18	164.5 (5)
C20—C16—C17—C18	0.5 (5)	C11—C12—Fe1—C18	45.0 (6)
Fe1—C16—C17—C18	60.3 (3)	C13—C12—Fe1—C16	−82.3 (3)
C20—C16—C17—Fe1	−59.8 (3)	C11—C12—Fe1—C16	158.1 (2)
C16—C17—C18—C19	−0.5 (5)	C13—C12—Fe1—C14	37.21 (19)
Fe1—C17—C18—C19	59.7 (3)	C11—C12—Fe1—C14	−82.34 (18)
C16—C17—C18—Fe1	−60.2 (3)	C11—C12—Fe1—C13	−119.6 (3)
C17—C18—C19—C20	0.2 (5)	C17—C18—Fe1—C19	118.8 (5)
Fe1—C18—C19—C20	60.2 (3)	C19—C18—Fe1—C17	−118.8 (5)
C17—C18—C19—Fe1	−59.9 (3)	C17—C18—Fe1—C20	81.3 (3)
C18—C19—C20—C16	0.1 (5)	C19—C18—Fe1—C20	−37.5 (3)
Fe1—C19—C20—C16	60.5 (3)	C17—C18—Fe1—C15	−123.9 (3)
C18—C19—C20—Fe1	−60.4 (3)	C19—C18—Fe1—C15	117.3 (3)
C17—C16—C20—C19	−0.3 (5)	C17—C18—Fe1—C11	−166.8 (3)
Fe1—C16—C20—C19	−60.2 (3)	C19—C18—Fe1—C11	74.4 (3)
C17—C16—C20—Fe1	59.9 (3)	C17—C18—Fe1—C12	158.7 (5)
C20—C19—Fe1—C17	−81.8 (3)	C19—C18—Fe1—C12	39.9 (7)
C18—C19—Fe1—C17	37.1 (3)	C17—C18—Fe1—C16	37.2 (3)
C18—C19—Fe1—C20	119.0 (4)	C19—C18—Fe1—C16	−81.6 (3)
C20—C19—Fe1—C15	160.1 (2)	C17—C18—Fe1—C14	−81.7 (4)
C18—C19—Fe1—C15	−80.9 (3)	C19—C18—Fe1—C14	159.5 (3)
C20—C19—Fe1—C11	116.9 (3)	C17—C18—Fe1—C13	−47.1 (6)
C18—C19—Fe1—C11	−124.2 (3)	C19—C18—Fe1—C13	−166.0 (4)
C20—C19—Fe1—C12	75.2 (3)	C17—C16—Fe1—C19	−79.7 (3)
C18—C19—Fe1—C12	−165.9 (3)	C20—C16—Fe1—C19	37.5 (3)
C20—C19—Fe1—C18	−119.0 (4)	C20—C16—Fe1—C17	117.2 (4)
C20—C19—Fe1—C16	−38.1 (3)	C17—C16—Fe1—C20	−117.2 (4)
C18—C19—Fe1—C16	80.8 (3)	C17—C16—Fe1—C15	41.9 (7)
C20—C19—Fe1—C14	−166.6 (3)	C20—C16—Fe1—C15	159.1 (4)
C18—C19—Fe1—C14	−47.6 (5)	C17—C16—Fe1—C11	−161.4 (4)
C20—C19—Fe1—C13	42.8 (6)	C20—C16—Fe1—C11	−44.1 (5)
C18—C19—Fe1—C13	161.8 (5)	C17—C16—Fe1—C12	161.7 (3)
C18—C17—Fe1—C19	−37.7 (3)	C20—C16—Fe1—C12	−81.0 (3)
C16—C17—Fe1—C19	82.3 (3)	C17—C16—Fe1—C18	−36.4 (3)
C18—C17—Fe1—C20	−81.3 (3)	C20—C16—Fe1—C18	80.9 (3)
C16—C17—Fe1—C20	38.8 (3)	C17—C16—Fe1—C14	77.4 (4)
C18—C17—Fe1—C15	74.8 (4)	C20—C16—Fe1—C14	−165.4 (3)
C16—C17—Fe1—C15	−165.1 (3)	C17—C16—Fe1—C13	119.0 (3)
C18—C17—Fe1—C11	35.8 (7)	C20—C16—Fe1—C13	−123.7 (3)
C16—C17—Fe1—C11	155.9 (4)	C15—C14—Fe1—C19	−46.5 (4)
C18—C17—Fe1—C12	−163.2 (3)	C13—C14—Fe1—C19	−166.5 (4)
C16—C17—Fe1—C12	−43.1 (6)	C15—C14—Fe1—C17	−121.9 (3)
C16—C17—Fe1—C18	120.1 (5)	C13—C14—Fe1—C17	118.2 (3)
C18—C17—Fe1—C16	−120.1 (5)	C15—C14—Fe1—C20	163.2 (5)
C18—C17—Fe1—C14	117.0 (3)	C13—C14—Fe1—C20	43.3 (6)
C16—C17—Fe1—C14	−122.9 (3)	C13—C14—Fe1—C15	−119.9 (3)
C18—C17—Fe1—C13	159.8 (3)	C15—C14—Fe1—C11	38.35 (17)
C16—C17—Fe1—C13	−80.1 (3)	C13—C14—Fe1—C11	−81.59 (19)
C16—C20—Fe1—C19	−118.6 (4)	C15—C14—Fe1—C12	82.67 (19)
C19—C20—Fe1—C17	80.1 (3)	C13—C14—Fe1—C12	−37.27 (18)
C16—C20—Fe1—C17	−38.4 (3)	C15—C14—Fe1—C18	−80.4 (3)
C19—C20—Fe1—C15	−45.5 (5)	C13—C14—Fe1—C18	159.7 (2)
C16—C20—Fe1—C15	−164.0 (3)	C15—C14—Fe1—C16	−163.6 (2)
C19—C20—Fe1—C11	−80.6 (3)	C13—C14—Fe1—C16	76.5 (3)
C16—C20—Fe1—C11	160.8 (3)	C15—C14—Fe1—C13	119.9 (3)
C19—C20—Fe1—C12	−123.9 (3)	C12—C13—Fe1—C19	42.4 (6)
C16—C20—Fe1—C12	117.5 (3)	C14—C13—Fe1—C19	162.2 (5)
C19—C20—Fe1—C18	37.5 (3)	C12—C13—Fe1—C17	158.7 (3)
C16—C20—Fe1—C18	−81.1 (3)	C14—C13—Fe1—C17	−81.5 (3)
C19—C20—Fe1—C16	118.6 (4)	C12—C13—Fe1—C20	74.9 (3)
C19—C20—Fe1—C14	161.8 (4)	C14—C13—Fe1—C20	−165.3 (2)
C16—C20—Fe1—C14	43.2 (6)	C12—C13—Fe1—C15	−82.6 (2)
C19—C20—Fe1—C13	−165.0 (2)	C14—C13—Fe1—C15	37.16 (17)
C16—C20—Fe1—C13	76.4 (3)	C12—C13—Fe1—C11	−37.86 (18)
C14—C15—Fe1—C19	160.0 (2)	C14—C13—Fe1—C11	81.94 (19)
C11—C15—Fe1—C19	−80.8 (2)	C14—C13—Fe1—C12	119.8 (3)
C14—C15—Fe1—C17	78.5 (3)	C12—C13—Fe1—C18	−167.9 (4)
C11—C15—Fe1—C17	−162.4 (3)	C14—C13—Fe1—C18	−48.1 (5)
C14—C15—Fe1—C20	−167.8 (4)	C12—C13—Fe1—C16	116.6 (3)
C11—C15—Fe1—C20	−48.6 (4)	C14—C13—Fe1—C16	−123.6 (2)
C14—C15—Fe1—C11	−119.2 (3)	C12—C13—Fe1—C14	−119.8 (3)
C14—C15—Fe1—C12	−80.67 (19)	C9—C8—N1—C6	−170.2 (3)
C11—C15—Fe1—C12	38.50 (17)	C7—C8—N1—C6	13.5 (5)
C14—C15—Fe1—C18	118.3 (2)	C5—C6—N1—C8	−132.8 (3)
C11—C15—Fe1—C18	−122.5 (2)	C1—C6—N1—C8	52.1 (5)
C14—C15—Fe1—C16	46.8 (6)		

### Hydrogen-bond geometry (Å, °)

**Table:**

*D*—H···*A*	*D*—H	H···*A*	*D*···*A*	*D*—H···*A*
N1—H1N···O1	0.85 (3)	1.94 (3)	2.632 (3)	139 (3)
O2—H2···O1^i^	0.87 (4)	1.79 (4)	2.662 (3)	178 (5)
C12—H12···O2^ii^	0.93	2.46	3.281 (4)	148

Symmetry codes: (i) *x*, −*y*+3/2, *z*−1/2; (ii) −*x*+1, −*y*+1, −*z*+1.
